# Netazepide Inhibits Expression of Pappalysin 2 in Type 1 Gastric Neuroendocrine Tumors

**DOI:** 10.1016/j.jcmgh.2020.01.010

**Published:** 2020-01-28

**Authors:** Katie A. Lloyd, Bryony N. Parsons, Michael D. Burkitt, Andrew R. Moore, Stamatia Papoutsopoulou, Malcolm Boyce, Carrie A. Duckworth, Klaire Exarchou, Nathan Howes, Lucille Rainbow, Yongxiang Fang, Claus Oxvig, Steven Dodd, Andrea Varro, Neil Hall, D. Mark Pritchard

**Affiliations:** 1 Department of Cellular and Molecular Physiology, Institute of Translational Medicine, University of Liverpool, Liverpool, United Kingdom; 4 Centre for Genomic Research, Institute of Integrative Biology, University of Liverpool, Liverpool, United Kingdom; 2 Liverpool University Hospitals, National Health Service Foundation Trust, Liverpool, United Kingdom; 3 Trio Medicines, Ltd, Hammersmith Medicines Research, London, United Kingdom; 5 Department of Molecular Biology and Genetics, Aarhus University, Aarhus C, Denmark; 6 The Earlham Institute, Norwich, Norfolk, United Kingdom; 7 School of Biological Sciences, University of East Anglia, Norwich, Norfolk, United Kingdom

**Keywords:** Tumorigenesis, Carcinogenesis, Mouse Model, Hormone, Signal Transduction, BSA, bovine serum albumin, CCK2R, cholecystokinin type-2 receptor, CHGA, chromogranin A, ECL, enterochromaffin-like, gNET, gastric neuroendocrine tumor, HDC, histidine decarboxylase, IGF, insulin-like growth factor, IGFBP, insulin-like growth factor binding protein, MMP, matrix metalloproteinase, mRNA, messenger RNA, PAPPA2, pappalysin 2, PBS, phosphate-buffered saline, qPCR, quantitative polymerase chain reaction, siRNA, small interfering RNA, TIMP, tissue inhibitors of metalloproteinases

## Abstract

**Background & Aims:**

In patients with autoimmune atrophic gastritis and achlorhydria, hypergastrinemia is associated with the development of type 1 gastric neuroendocrine tumors (gNETs). Twelve months of treatment with netazepide (YF476), an antagonist of the cholecystokinin B receptor (CCKBR or CCK2R), eradicated some type 1 gNETs in patients. We investigated the mechanisms by which netazepide induced gNET regression using gene expression profiling.

**Methods:**

We obtained serum samples and gastric corpus biopsy specimens from 8 patients with hypergastrinemia and type 1 gNETs enrolled in a phase 2 trial of netazepide. Control samples were obtained from 10 patients without gastric cancer. We used amplified and biotinylated sense-strand DNA targets from total RNA and Affymetrix (Thermofisher Scientific, UK) Human Gene 2.0 ST microarrays to identify differentially expressed genes in stomach tissues from patients with type 1 gNETs before, during, and after netazepide treatment. Findings were validated in a human AGS_GR_ gastric adenocarcinoma cell line that stably expresses human CCK2R, primary mouse gastroids, transgenic hypergastrinemic INS-GAS mice, and patient samples.

**Results:**

Levels of pappalysin 2 (*PAPPA2*) messenger RNA were reduced significantly in gNET tissues from patients receiving netazepide therapy compared with tissues collected before therapy. PAPPA2 is a metalloproteinase that increases the bioavailability of insulin-like growth factor (IGF) by cleaving IGF binding proteins (IGFBPs). PAPPA2 expression was increased in the gastric corpus of patients with type 1 gNETs, and immunohistochemistry showed localization in the same vicinity as CCK2R-expressing enterochromaffin-like cells. Up-regulation of PAPPA2 also was found in the stomachs of INS-GAS mice. Gastrin increased PAPPA2 expression with time and in a dose-dependent manner in gastric AGS_GR_ cells and mouse gastroids by activating CCK2R. Knockdown of PAPPA2 in AGS_GR_ cells with small interfering RNAs significantly decreased their migratory response and tissue remodeling in response to gastrin. Gastrin altered the expression and cleavage of IGFBP3 and IGFBP5.

**Conclusions:**

In an analysis of human gNETS and mice, we found that gastrin up-regulates the expression of gastric PAPPA2. Increased PAPPA2 alters IGF bioavailability, cell migration, and tissue remodeling, which are involved in type 1 gNET development. These effects are inhibited by netazepide.

## Graphical abstract

**Figure:**
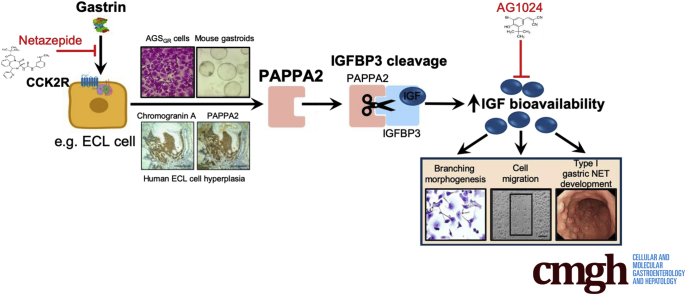

**Summary**

Expression of the metalloproteinase pappalysin 2 was inhibited in the stomach of patients with type I gastric neuroendocrine tumors after treatment with the gastrin/cholecystokinin 2 receptor antagonist, netazepide. Gastrin-induced pappalysin 2 expression in cholecystokinin 2R–expressing gastric epithelial cells resulted in increased insulin-like growth factor bioavailability, promotion of cell migration, and tissue remodeling.

Gastric neuroendocrine (carcinoid) tumors (gNETs) are relatively rare and originate from enterochromaffin-like (ECL) cells in the oxyntic mucosa of the stomach. They are classified as 3 types (with an extremely rare fourth type) on the basis of pathologic and morphologic characteristics.^1^^,^^2^ Type 1 gNETs develop as a consequence of the hypergastrinemia that is associated with autoimmune atrophic gastritis, achlorhydria, and frequently pernicious anemia. Although type 1 gNETs usually are grade 1 (Ki67 proliferative index, <2%) and frequently have an indolent clinical course, 1%–20% of patients develop metastases.^3^^,^^4^

The physiological functions of the hormone gastrin have been investigated largely within the stomach, focusing primarily on acid secretion after cholecystokinin type-2 receptor (CCK2R) activation.^5^ However, gastrin also plays a central role in regulating gastric tissue remodeling and cell migration.^5^, ^6^, ^7^, ^8^, ^9^, ^10^ These changes are thought to play a role in type 1 gNET development.

Previous studies have suggested that matrix metalloproteinases (MMPs) and insulin-like growth factors (IGFs) play important roles in regulating cellular pathways and thus tumor development in the stomach. Infection with *Helicobacter pylori* increases the production and secretion of MMP7 from gastric epithelial cells.^11^, ^12^, ^13^ Secreted MMP7 liberates IGF-II from IGF binding protein 5 (IGFBP-5) (which is released from subepithelial cells) and stimulates the expansion and migration of cells in the surrounding gastric microenvironment.^14^^,^^15^ Hypergastrinemia also increases gastric MMP7 expression (as well as that of MMP1^7^ and MMP9^9^), and this is thought to promote type 1 gNET development via a similar mechanism.^10^^,^^14^^,^^15^

Small localized type 1 gNETs often can be successfully removed endoscopically.^2^ However, in many cases, complete endoscopic resection is not possible owing to polyp multiplicity. Therefore, other methods of treatment sometimes need to be considered. Antrectomy can be effective by removing gastrin-secreting G cells,^16^ but this involves invasive surgery. Small case series also have reported benefits from using long-acting somatostatin analogues.^17^^,^^18^ However, most recently, attention has been given to the potential role of a gastrin/CCK2R antagonist.

Netazepide (YF476) at a concentration of 500 μmol/kg has been shown to inhibit ECL cell hyperproliferation and spontaneous type 1 gNET development in African cotton rats (*Sigmodon hispidus*)^19^ and *Mastomys* rodents (*Praomys natalensis*).^20^ Recent clinical trials also have shown that patients with type 1 gNETs who received a single daily dose of netazepide orally showed significant reductions in gNET size and number at both 12 weeks and 12 months.^21^, ^22^, ^23^ Biomarkers of gastric pathology such as chromogranin A (a biomarker of ECL cell activity), MMP7, and histidine decarboxylase (HDC) also were suppressed during therapy, whereas serum gastrin concentrations remained unaffected.

The mechanisms by which netazepide induces these effects in type 1 gNET patients, however, currently are unknown. We therefore performed a transcriptomic study using gastric biopsy samples obtained from patients before, during, and after netazepide treatment to investigate the molecular pathways that were altered during therapy with this drug. One of the messenger RNAs (mRNAs) that showed significant changes in expression was pappalysin 2 (PAPPA2). Because this protein is known to promote IGF bioavailability in other tissues,^24^ and because IGFs have a proven involvement in gastric tumorigenesis,^14^^,^^15^ we concentrated our subsequent analyses on this protein.

## Results

### Netazepide Alters the Expression of Several Genes in Hypergastrinemic Patients With Type 1 gNETs

**Figure 1:**
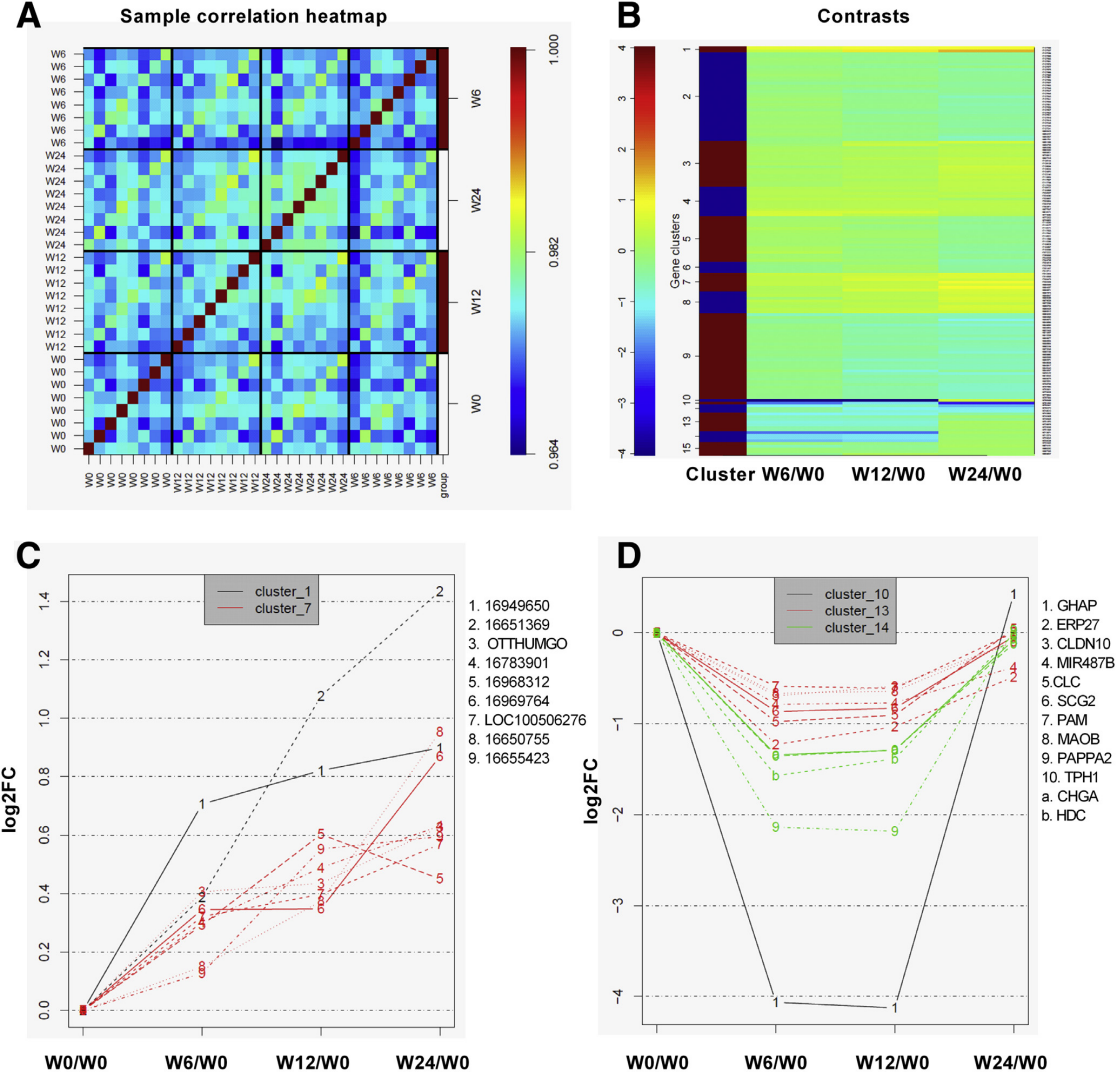
**(*A*) Correlation heatmap of expression levels among all samples and (*B*) heatmap of the clustered log_2_ fold change (FC) for differentially expressed genes**. The 15 clusters are indicated by the *red bars* and *blue bars*. A gene’s FC in expression level relative to time point week 0 (W0) is indicated by heatmap colors. Each *row* represents a gene, and regulation profile plot of significant clusters is expressed as FC. (*C*) The expression of clusters 1 and 7 continued to increase after withdrawal of netazepide, and the expression of clusters 10, 13, and 14 decreased while the patients were taking netazepide but returned to pretreatment levels after cessation of the drug. W0, week 0/baseline; W6, 6 weeks on netazepide; W12, 12 weeks on netazepide; W24, 12 weeks after cessation of netazepide treatment. CHGA, Chromogranin A; CLC, Charcot-Leyden Crystal Galectin; CLDN10, Claudin 10; ERP27, Endoplasmic reticulum protein 27; GHAP, Glial Hyaluronate-binding protein; GHAP, Glial Hyaluronate-binding protein; HDC, Histidine Decarboxylase; CHGA, Chromogranin A; MAOBMonoamine Oxidas, e B; MIR487B, miRNA487b; PAM, Peptidyl alpha-amidating monooxygenase; PAPPA2, Pregnancy-associated plasma protein A2; SCG2, Secretogranin II; TPH1, Tryptophan Hydroxylase 1.

Microarray analysis identified several clusters of genes that were expressed differentially in the gastric corpus of hypergastrinemic patients with type 1 gNETs taken before, during (6 and 12 weeks), and 12 weeks after a 50-mg oral daily dose of netazepide (Figure 1*A* and 1*B*). Of these, clusters 1 and 7 increased after withdrawal of netazepide (Figure 1*C*), and clusters 10, 13, and 14 showed a significant decrease in expression while patients were taking netazepide (weeks 6 and 12), which returned to pretreatment levels after treatment withdrawal (Figure 1*D*). Netazepide-inhibited genes within this group including the following: glycoprotein hormones α polypeptide, endoplasmic reticulum protein 27, claudin-10, miRNA-487b, Charcot–Leyden crystal galectin, secretogranin II, peptidyl-glycine α-amidating mono-oxygenase, monoamine oxidase B, *PAPPA2*, tryptophan hydroxylase 1, chromogranin A (*CHGA*), and *HDC*. A list of the most up-regulated and down-regulated genes after 12 weeks of netazepide relative to baseline is shown in Supplementary Table 1. We previously reported significant decreases in the gastric mRNA expression of CHGA and HDC during netazepide treatment.^22^ Of the other genes, the highest fold changes were observed with glycoprotein hormones α polypeptide and PAPPA2. Because PAPPA2 is a metalloproteinase that regulates the IGF pathway,^24^ which is known to be involved in gNET development, this gene/protein was chosen for further investigation.

### PAPPA2 Expression Increases in the Stomach, but Not the Serum, of Hypergastrinemic Patients With Type 1 gNETs

**Figure 2:**
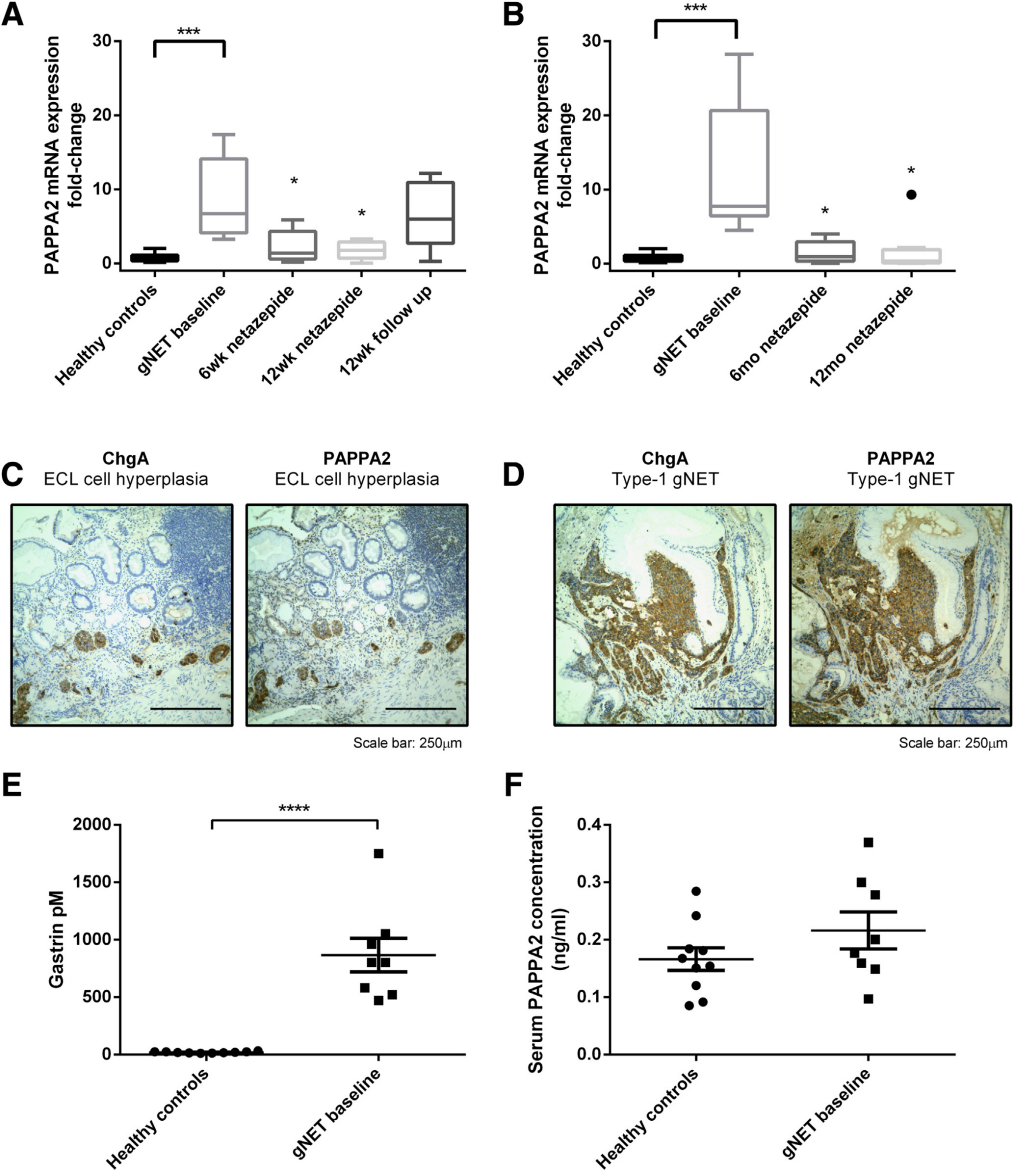
**PAPPA2 mRNA expression was confirmed using qPCR in 8 patients with type 1 gNETs and 10 healthy control subjects**. PAPPA2 mRNA expression decreased significantly while the gNET patients were taking a 50-mg oral daily dose of netazepide and returned to baseline after cessation of treatment in (*A*) 12-week and (*B*) 12-month studies. Statistical significance was determined using the Mann–Whitney *U* test between independent healthy controls and baseline samples and a Wilcoxon signed-rank test between repeated samples with Bonferroni correction for multiple comparisons because not all the samples were distributed normally. *P* < .0125 was considered significant after Bonferroni correction. ∗*P* < .0125, ∗∗∗*P* < .001, and ∗∗∗∗*P* < .0001. Immunohistochemical staining showed increased PAPPA2 and ChgA protein expression in serial histologic sections of (*C*) human micronodular ECL cell hyperplasia and (*D*) type 1 gNET tumor tissues. However, no significant differences in circulating PAPPA2 protein concentrations were observed between (*E*) patients with hypergastrinemia and type 1 gNETs (n = 8) compared with (*F*) healthy controls (n = 10).

In gastric corpus biopsy specimens taken from 8 patients with hypergastrinemia and type 1 gNETs, PAPPA2 mRNA abundance was significantly higher at baseline compared with biopsy specimens from 10 healthy normogastrinemic control subjects. Gastric PAPPA2 mRNA expression decreased while patients were taking 50-mg oral daily netazepide, both in the short-term 12 week (Figure 2*A*) and long-term 12-month studies (Figure 2*B*). These data supported the previous microarray findings and also confirmed sustained inhibition of gastric PAPPA2 mRNA expression by netazepide in the longer 12-month trial.

**Figure 3:**
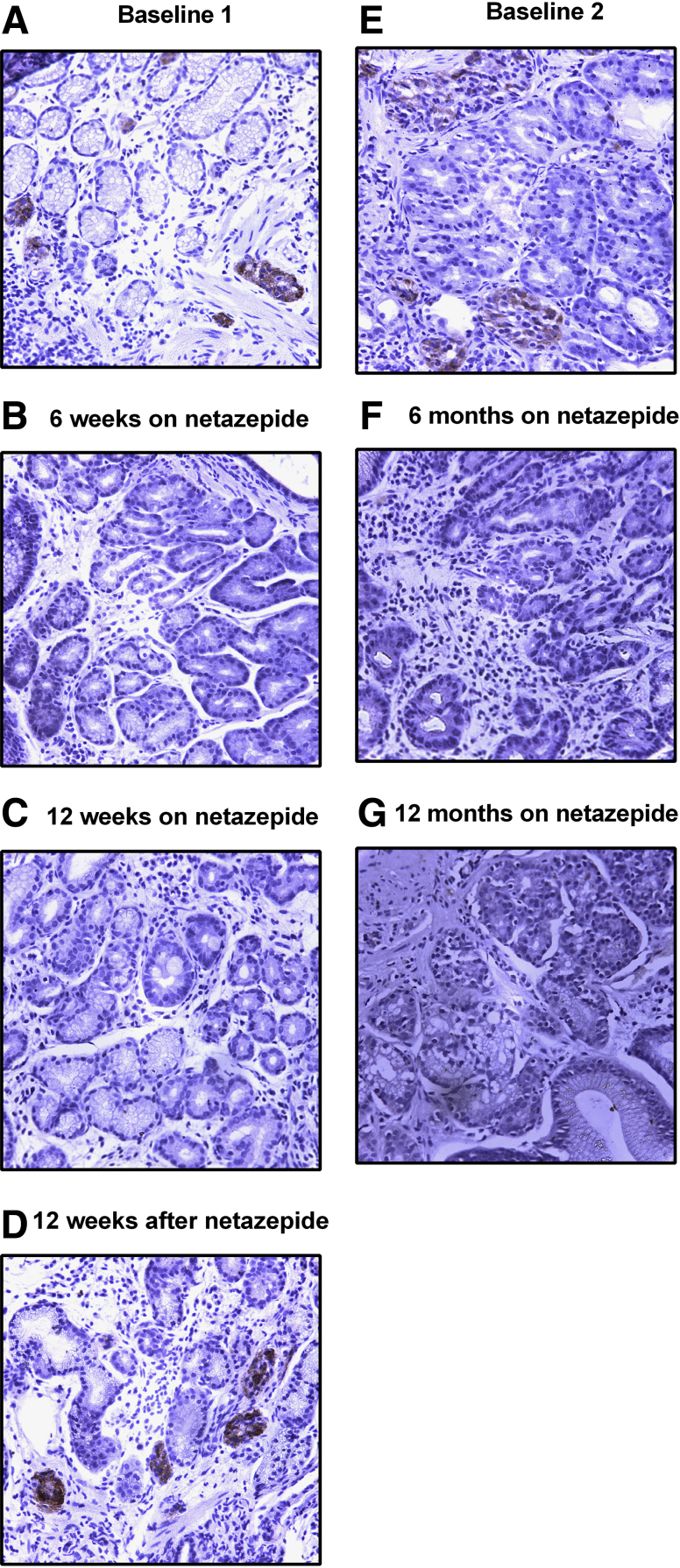
**Representative immunohistochemical staining for PAPPA2 in human gastric corpus biopsy specimens from a patient who was taking netazepide for 12 weeks (*A-C*) with a 12-week follow-up evaluation (*D*), and then the same patient on netazepide for a further 12 months (*E-G*)**. Increased PAPPA2 staining was observed only in samples when the patient was not taking netazepide (*A, D, E*).

Immunohistochemical analysis of serial sections indicated localization of PAPPA2 in areas of the tissue that also expressed chromogranin A, thus representing areas of micronodular ECL cell hyperplasia (Figure 2*C*) and type 1 gNETs (Figure 2*D*) in human gastric biopsy specimens, suggesting that this protein is specifically up-regulated in CCK2R-expressing gNET cells. However, circulating PAPPA2 concentrations showed no significant differences between hypergastrinemic type 1 gNET patients and healthy controls (Figure 2*E* and *F*). Immunohistochemical PAPPA2 expression was not detected in gastric corpus biopsy specimens taken from the same patient while taking netazepide (Figure 3). These data therefore suggest that gastrin increases the expression of PAPPA2 locally in the gastric mucosa, but the increased expression appears not to be reflected significantly in the circulation.

### Gastrin Increases PAPPA2 mRNA and Protein Expression in AGS_GR_ Cells via the CCK_2_ Receptor

To investigate whether gastrin directly affects PAPPA2 expression in CCK2R-expressing gastric epithelial cells, we used human gastric adenocarcinoma cells that have been stably transfected with the human CCK_2_ receptor (AGS_GR_).

**Figure 4:**
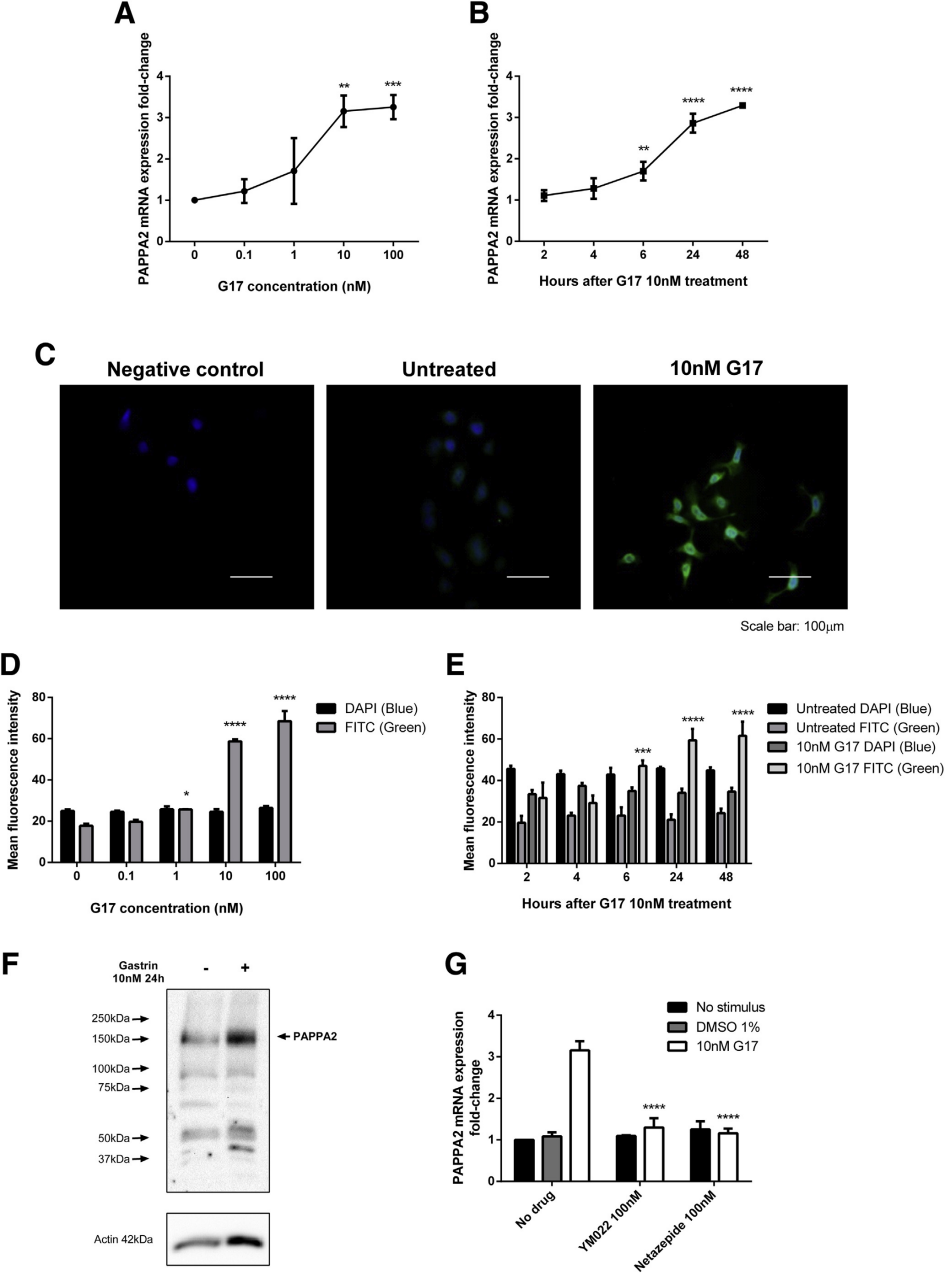
**(*A* and *B*) PAPPA2 mRNA and (*C–D*) protein expression were assessed using qPCR and immunofluorescence, respectively, and showed increases in expression in dose- and time-dependent manners after gastrin treatment**. Maximal increases were observed after 10 nmol/L gastrin for 24 hours. (*F*) Western blot for PAPPA2 in AGS_GR_ cells treated with and without 10 nmol/L gastrin for 24 hours. (*G*) Gastrin-stimulated PAPPA2 mRNA expression was completely reversed after pretreatment with CCK2R antagonists YM022 (100 nmol/L) or netazepide (100 nmol/L). Statistical significance was determined using either 1-way or 2-way analysis of variance where appropriate with the Sidak post hoc test, and *P* < .05 was considered significant. ∗*P* < .05, ∗∗*P* < .01, ∗∗∗*P* < .001, and ∗∗∗∗*P* < .0001 vs untreated control at the same time point. Densitometry was performed using AxioVision Rel. 4.8 with a mean number of 132 ± 13 cells analyzed per treatment. DAPI, 3,3′-diaminobenzidine tetra hydrochloride; FITC, fluorescein isothiocyanate.

PAPPA2 mRNA abundance dose-dependently (Figure 4*A*) and time-dependently (Figure 4*B*) increased with gastrin treatment (N = 3, n = 4), and was maximal after 10 nmol/L gastrin for 24 hours. Immunocytochemistry (representative images are shown in Figure 4*C*) also showed that PAPPA2 protein expression increased significantly in AGS_GR_ cells in a dose- and time-dependent manner after gastrin treatment (Figure 4*D* and *E*). Western blot confirmed this increase (Figure 4*F*). Pretreatment with the CCK_2_-receptor antagonist YM022 or netazepide (both at 100 nmol/L) completely reversed the increased expression of PAPPA2 caused by 10 nmol/L gastrin for 24 hours (Figure 4*G*).

### Gastrin Stimulates Cell Growth and Increases PAPPA2 Expression in Mouse Gastric Organoids via the CCK_2_ Receptor

**Figure 5:**
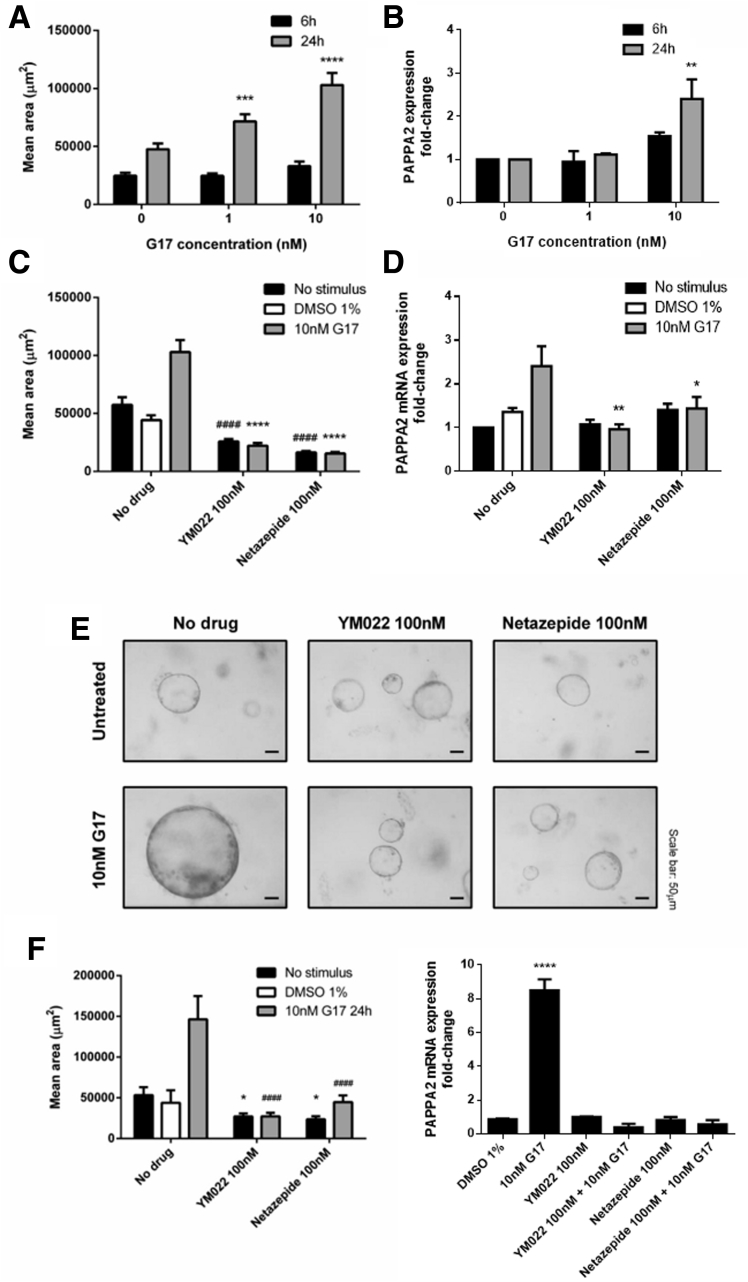
**Gastrin treatment increased the (*A*) mouse gastric organoid area (μm^2^) and (*B*) PAPPA2 mRNA expression and was maximal after 10 nmol/L gastrin for 24 hours**. (*C* and *D*) The increased organoid area and PAPPA2 mRNA expression caused by 10 nmol/L gastrin were completely reversed by pretreatment with CCK2R antagonist drugs YM022 or netazepide (both 100 nmol/L). (*E*) Representative bright-field images were taken after 10 nmol/L gastrin treatment for 24 hours with and without YM022 or netazepide pretreatment. Gastrin treatment also increased transgenic INS-GAS mouse-derived gastric organoid area (μm^2^) and PAPPA2 mRNA expression after 10 nmol/L G17 for 24 hours. The increased organoid area and PAPPA2 mRNA expression caused by 10 nmol/L G17 was completely reversed by pretreatment with CCK2R antagonist drugs YM022 or netazepide (both 100 nmol/L). Statistical significance was determined using 2-way analysis of variance with the Sidak post hoc test and ∗*P* < .05 was considered significant. ∗∗*P* < .01, ∗∗∗*P* < .001, and ∗∗∗∗*P* < .0001 vs 10 nmol/L gastrin positive control and ^####^*P* < .0001 vs untreated control. DMSO, dimethyl sulfoxide.

**Figure 6:**
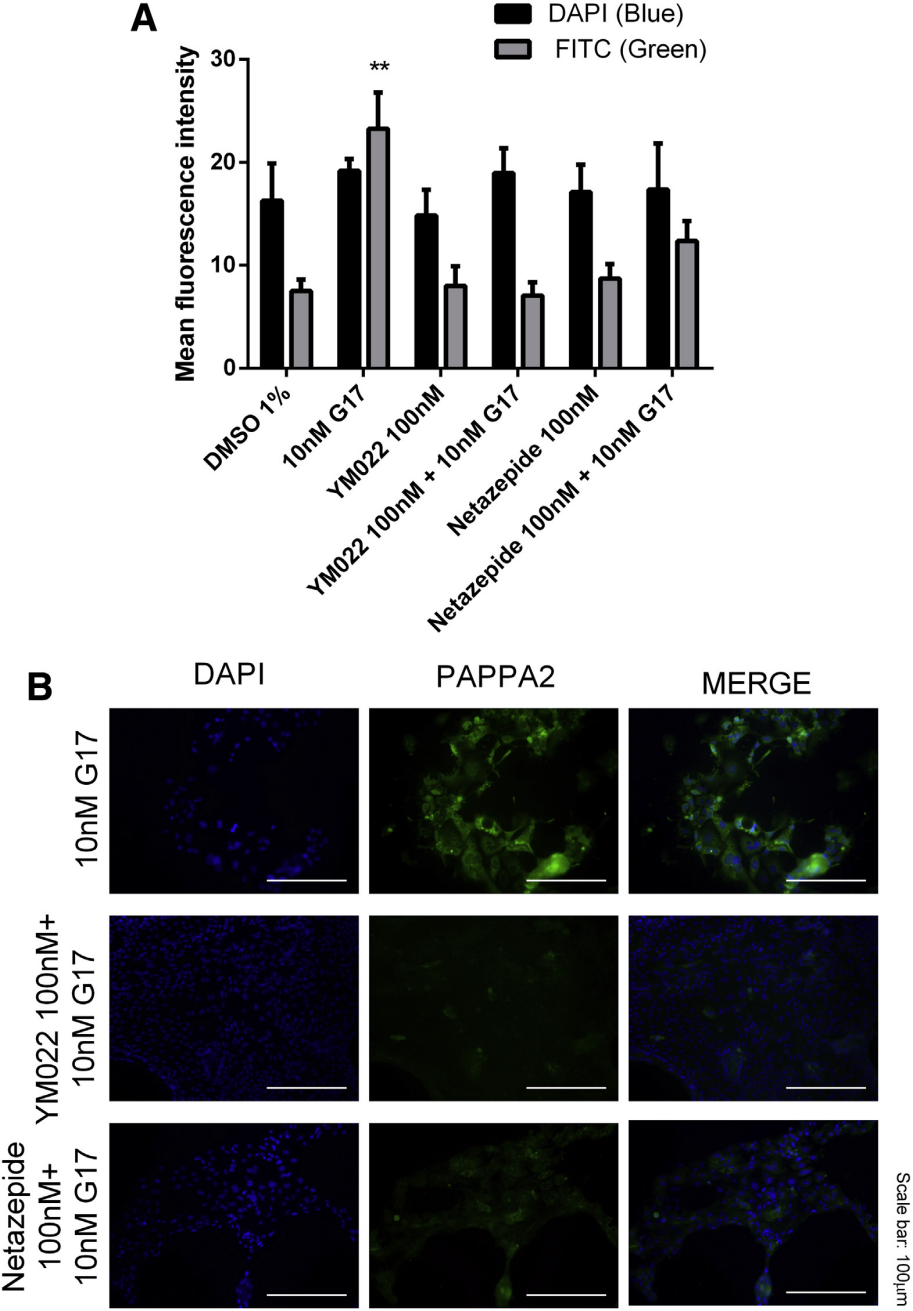
**Pretreatment with either YM022 or netazepide at 100 nmol/L significantly reduced gastrin-induced PAPPA2 protein expression in 2-dimensional primary cell cultures derived from wild-type mouse gastric organoids.** (*A*) Densitometry was performed using AxioVision Rel. 4.8 on 3 reference fields per treatment. (*B*) Representative images were taken per treatment. Statistical significance was determined using 2-way analysis of variance with the Sidak post hoc test and *P* < .05 was considered significant. ∗∗*P* < .01 vs vehicle only control (DMSO 1%). DAPI, 3,3′-diaminobenzidine tetra hydrochloride; DMSO, dimethyl sulfoxide; FITC, fluorescein isothiocyanate.

To investigate the effect of gastrin on PAPPA2 expression in the context of the mixed cell population of the gastric epithelium, primary mouse gastric organoid cultures were used.^25^ Gastrin treatment increased the average gastric organoid area in a dose- and time-dependent manner. This was significant after 1 nmol/L gastrin for 24 hours and maximal after 10 nmol/L gastrin for 24 hours (Figure 5*A*). Gastrin treatment also dose- and time-dependently increased PAPPA2 mRNA expression and this was significant after 10 nmol/L gastrin for 24 hours (Figure 5*B*) (N = 3, n = 4). A similar increase in immunofluorescent PAPPA2 expression also was observed after gastrin treatment (Figure 6).

Both gastrin-stimulated gastric organoid growth and increased PAPPA2 expression were completely reversed by pretreatment with CCK2R antagonist drugs YM022 or netazepide (both at 100 nmol/L) (Figure 5*C* and *D*) (N = 3, n = 4). Representative bright-field images are shown of mouse gastric organoids with and without pretreatment with CCK2R antagonist drugs YM022 or netazepide (both at 100 nmol/L), and with and without 10 nmol/L gastrin treatment for 24 hours (Figure 5*E*). Similar observations in response to gastrin and CCK2R antagonists also were made in gastroids derived from INS-GAS mice (Figure 5*F*).

### PAPPA2 Gene Expression Is Increased Significantly in the Stomach of Hypergastrinemic INS-GAS Mice

**Figure 7:**
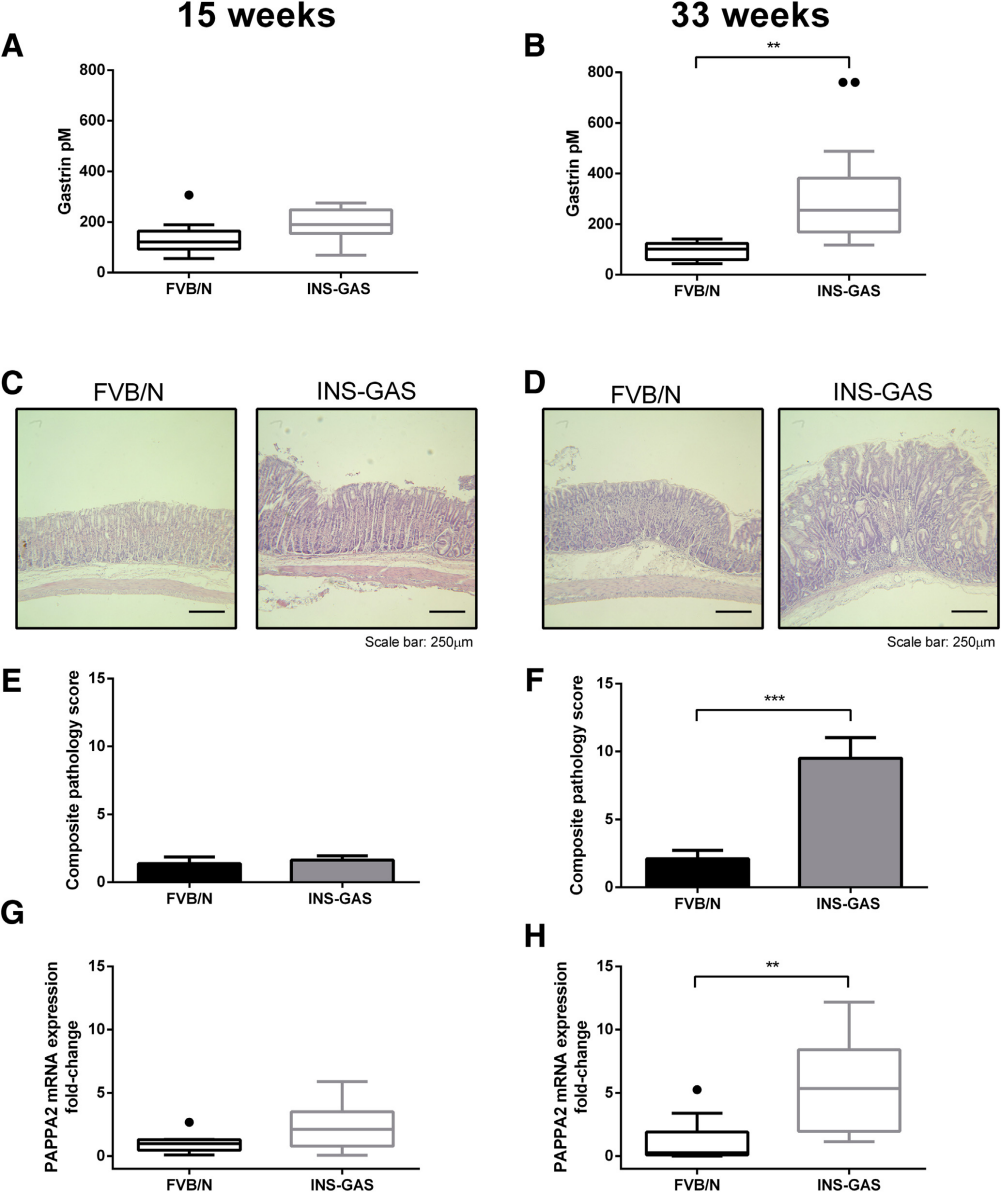
**(*A* and *B*) Circulating serum gastrin concentrations increased with age in transgenic INS-GAS mice and were increased significantly at 33 weeks of age compared with age-matched FVB/N control mice**. (*C*) Corpus histology showed no significant morphologic changes in 15-week INS-GAS mice compared with age-matched FVB/N controls. (*D*) However, extensive gastric tissue remodeling was observed in the corpus of 33-week-old INS-GAS mice compared with age-matched wild-type mice. (*E* and *F*) PAPPA2 mRNA expression was increased significantly in INS-GAS mice at 33 weeks, but not 15 weeks of age compared with age-matched FVB/N controls. Statistical significance was determined using Student *t* tests and *P* < .05 was considered significant (n = 10 mice per group). ∗∗*P* < .01 and ∗∗∗*P* < .001.

Gastrin radioimmunoassay showed no significant differences in serum gastrin concentrations between 15-week-old transgenic INS-GAS mice and age- and sex-matched FVB/N controls (Figure 7*A*). However, circulating gastrin concentrations increased with age in the INS-GAS mice and were increased significantly compared with age-matched wild-type FVB/N mice at 33 weeks of age (Figure 7*B*).

Histologic analysis confirmed minimal hyperplasia, but no other significant structural differences in the corpus of INS-GAS mice compared with age-matched FVB/N controls at 15 weeks of age (Figure 7*C*). However, as expected and previously described,^26^ remodeling of the gastric corpus mucosa with hyperplasia, atrophy, and loss of parietal cells was observed in 33-week-old hypergastrinemic INS-GAS mice compared with normogastrinemic age-matched FVB/N wild-type controls (Figure 7*D*).

Quantitative polymerase chain reaction (qPCR) analysis of gastric mucosal scrapes showed no significant differences in PAPPA2 mRNA between 15-week INS-GAS mice and wild-type FVB/N controls (Figure 7*E*). However, PAPPA2 mRNA expression was increased significantly in 33-week INS-GAS mice relative to age-matched, wild-type FVB/N mice, in keeping with the observed hypergastrinemia and altered gastric corpus histology at this age (Figure 7*F*).

### Gastrin-Stimulated PAPPA2 Expression Significantly Increases Cell Structural Remodeling and Migration

**Figure 8:**
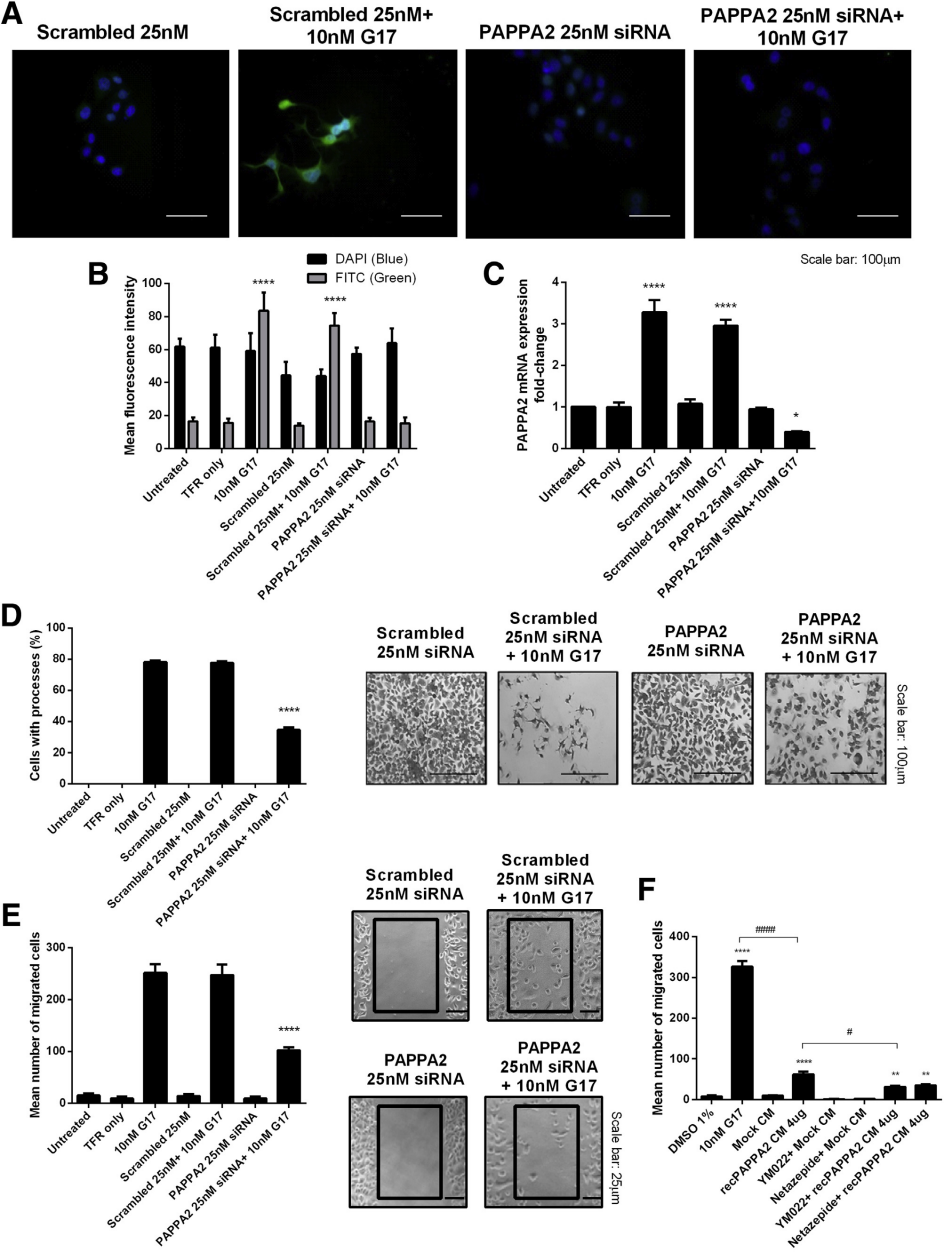
**Immunofluorescence and qPCR confirmed PAPPA2 (25 nmol/L) siRNA knockdown in AGS_GR_ cells after 48 hours of transfection, with a (*A* and *B*) >80% reduction in protein expression and a (*C*) >90% reduction in PAPPA2 mRNA**. Densitometry was performed using AxioVision Rel. 4.8 with a mean number of 147 ± 22 cells analyzed per treatment. PAPPA2 siRNA knockdown significantly reduced the (*D*) extension of long processes at 6 hours and (*E*) cell migration at 8 hours induced by 10 nmol/L gastrin treatment. Representative images show changes in (*D*) cell morphology and (*E*) migration assays. Statistical significance was determined using either 1-way or 2-way analysis of variance where appropriate with the Sidak post hoc test, and *P* < .05 was considered significant. ∗*P* < .05, and ∗∗∗∗*P* < .0001 vs scrambled (25 nmol/L) control at the same time point. (*F*) Recombinant PAPPA2 conditioned media (4 μg/mL) increased AGS_GR_ cell migration compared with untreated controls, but was significantly less than the 10 nmol/L G17 14-hour positive control. Statistical significance was determined using 1-way analysis of variance with the Sidak post hoc test, and *P* < .05 was considered significant. ∗∗*P* < .01 and ∗∗∗∗*P* < .0001 vs vehicle control, ^#^*P* < .05 and ^####^*P* < .0001. DAPI, 3,3′-diaminobenzidine tetra hydrochloride; DMSO, dimethyl sulfoxide; FITC, fluorescein isothiocyanate.

Immunofluorescence and qPCR were used initially to confirm the optimal experimental conditions for the successful knockdown of PAPPA2 mRNA and protein in AGS_GR_ cells. Quantification of PAPPA2 expression by immunofluorescence (Figure 8*A* and 8*B*) and qPCR (Figure 8*C*) showed that PAPPA2 small interfering RNA (siRNA) (25 nmol/L, for 48 h) successfully reduced gastrin-induced PAPPA2 mRNA and protein expression in AGS_GR_ cells by more than 80% and more than 90%, respectively (N = 3, n = 4). Scrambled siRNAs had no such effect.

We chose not to test the effects of PAPPA2 inhibition on cell proliferation because gastrin is known to directly inhibit rather than promote the proliferation of AGS_GR_ cells.^27^ Instead, we decided to investigate 2 other gastrin-induced cellular phenomena that are associated with tumor development.

Gastrin previously was shown to induce the remodeling of the actin cytoskeleton via the extension of long processes in AGS_GR_ cells.^6^ Gastrin (10 nmol/L for 6 h) induced the extension of long processes in AGS_GR_ cells. AGS_GR_ cells transfected with 25 nmol/L PAPPA2 siRNA for 48 hours showed a significant decrease in the proportion of cells showing the extension of long processes after treatment with 10 nmol/L gastrin for 6 hours (*P* < .0001). PAPPA2 siRNA (25 nmol/L) alone did not have any significant effect on cell morphology (N = 3, n = 3). Representative images are shown of AGS_GR_ cells transfected with PAPPA2 25 nmol/L siRNA with and without 10 nmol/L gastrin treatment for 6 hours to allow visual comparisons of cell morphology (Figure 8*D*).

Gastrin also previously has been shown to promote the migration of AGS_GR_ cells.^8^ Gastrin alone (10 nmol/L) stimulated AGS_GR_ cell migration after 8 hours. Transfection with PAPPA2 siRNA 25 nmol/L for 48 hours had no effect on AGS_GR_ cell migration. However, PAPPA2 siRNA at a concentration of 25 nmol/L for 48 hours significantly reduced the migratory response of AGS_GR_ cells after addition of 10 nmol/L gastrin treatment for 8 hours (*P* < .0001; N = 3, n = 3). Representative images are shown of the migration of AGS_GR_ cells transfected with PAPPA2 25 nmol/L siRNA with and without 10 nmol/L gastrin treatment for 8 hours (Figure 8*E*).

Conditioned media from PAPPA2-secreting cells also increased the migration of AGS_GR_ cells, but at the concentration tested the response was of a lower magnitude than that induced by gastrin (Figure 8*F*).

### Gastrin Increases AGS_GR_ Cell Migration and Cellular Remodeling in an IGF-Dependent Manner

Because PAPPA2 is known to promote the cleavage of IGFBPs, thus altering the bioavailability of IGFs, we investigated components of this signaling pathway using Western blot and the IGF-1–receptor inhibitor AG1024.

**Figure 9:**
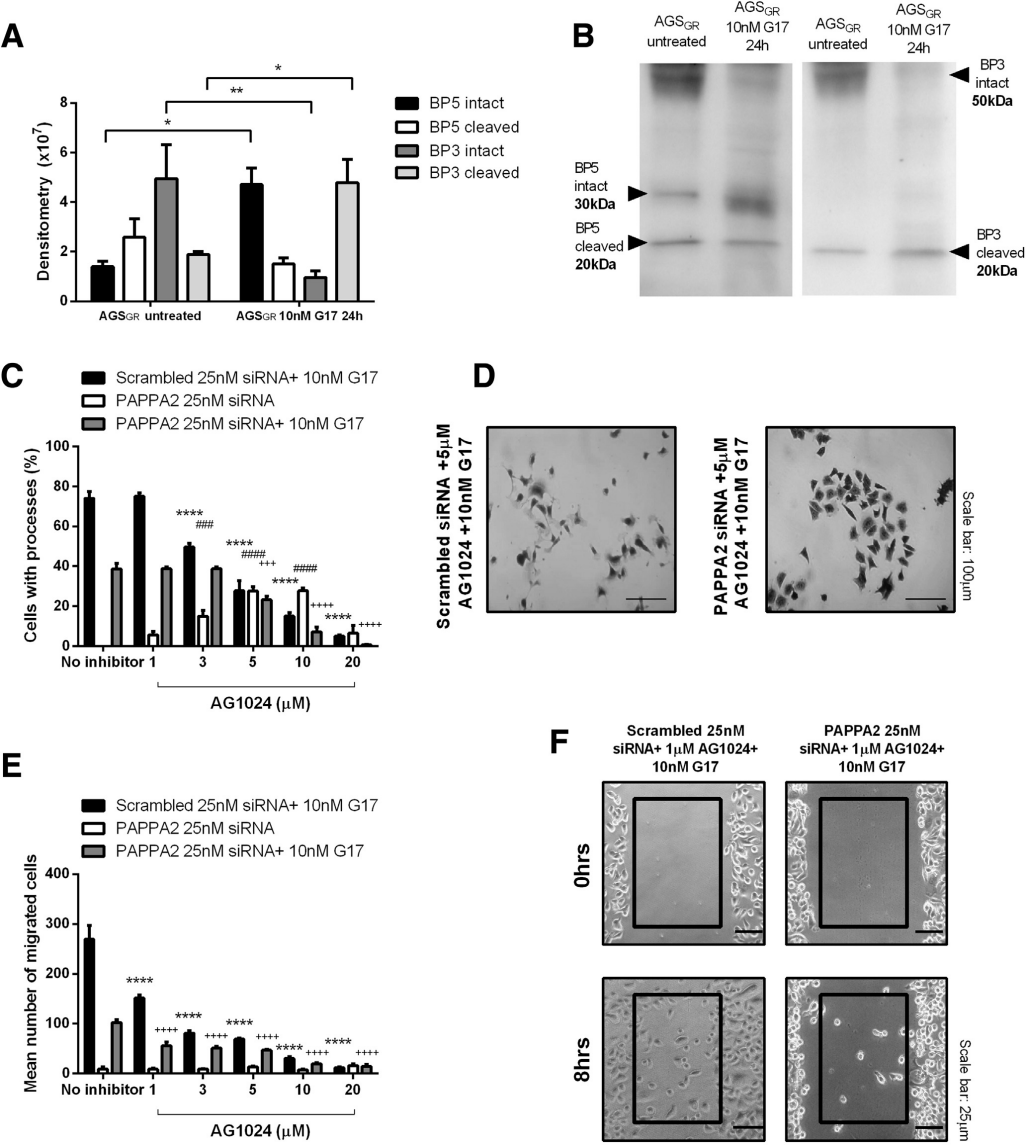
**(*A* and *B*) Gastrin treatment (10 nmol/L, 24 h) increased the expression of IGFBP-5 and cleavage of IGFBP-3 in the media of AGS_GR_ cells**. Experiments were performed in triplicate and statistical significance was determined using 2-way analysis of variance with the Sidak post hoc test. ∗*P* < .05 and ∗∗*P* < .01. Pretreatment (20 min) with the IGF inhibitor AG1024 further suppressed (*C* and *D*) gastrin-induced migration and (*E* and *F*) extension of long processes in AGS_GR_ cells that were transfected with 25 nmol/L PAPPA2 siRNA for 48 hours. Experiments were completed 3 times and statistical significance was determined using 2-way analysis of variance with the Sidak post hoc test. *P* < .05 was considered significant, ∗∗∗∗*P* < .0001 scrambled 25 nmol/L siRNA+ 10 nmol/L gastrin vs no inhibitor. ^###^*P* < .001 and ^####^*P* < .0001 PAPPA2 25 nmol/L siRNA vs no inhibitor, and ^+++^*P* < .001 and ^++++^*P* < .0001 PAPPA2 25 nmol/L siRNA +10 nmol/L gastrin vs no inhibitor.

A total of 10 nmol/L gastrin for 24 hours stimulated increased expression of intact IGFBP-5 (*P* < .05) and cleaved IGFBP-3 (*P* < .01) (Figure 9*A* and *B*) in the media of AGS_GR_ cells (n = 3). To investigate whether the increased cleavage of IGFBP-3 resulted in increased IGF bioavailability and to what extent this influenced gastrin-induced cellular migration and structural remodeling, we used the IGF-1–receptor inhibitor AG1024. In the presence of scrambled siRNA, pretreatment with 3–20 μmol/L AG1024 for 20 minutes followed by 10 nmol/L gastrin treatment for 6 hours resulted in significant decreases in the percentage of cells expressing long processes (*P* < .0001) (Figure 9*C* and *D*). Similarly, the cellular migration induced by 10 nmol/L gastrin for 8 hours was inhibited significantly by 1–20 μmol/L AG1024 (*P* < .0001) (Figure 9*E* and *F*). Transfection with PAPPA2 siRNA (25 nmol/L) for 48 hours resulted in significant partial (approximately 50%) decreases in gastrin-induced AGS_GR_ cell remodeling and migration, and both of these parameters were further and completely decreased by 10–20 μmol/L AG1024 pretreatment (*P* < .001 and *P* < .0001, respectively) (Figure 9*C–F*). These findings suggest that the actions of gastrin are wholly dependent on IGF-R, but that PAPPA2-mediated IGFBP cleavage may be partially responsible for this. For an unknown reason, AG1024 appeared to result in a small increase in the percentage of cells expressing long processes after transfection with PAPPAs siRNA, but in the absence of gastrin (Figure 9*C*).

## Discussion

We have shown that several genes showed reduced abundance in the stomach of hypergastrinemic type 1 gNET patients during treatment with the gastrin/CCK2R antagonist netazepide. Among these were 2 ECL cell–specific genes, CHGA and HDC, which we previously had shown by qPCR to be reduced in abundance in the gastric corpus during netazepide therapy.^22^^,^^23^ Of the other 10 genes that also showed significant reductions in abundance during netazepide therapy, several (such as secretogranin II and peptidyl-glycine α-amidating mono-oxygenase) already are known to be associated with the gastrin signaling pathway. Further investigation of all these netazepide-regulated genes eventually will be required to investigate the extent to which they individually contribute toward regulating a patient’s response to this drug.

However, we were intrigued to find that the abundance of the metalloproteinase PAPPA2 was reduced significantly in the stomach during netazepide treatment. PAPPA2 is known to promote IGF signaling, a critical pathway involved in gastric tumor development. However, PAPPA2 has not been associated previously with gastrointestinal disease, gastrin signaling, or neuroendocrine tumor development. We therefore focused the remainder of this current study on this protein.

We showed that PAPPA2 expression is increased in the gastric mucosa of hypergastrinemic human subjects (Figure 2) and transgenic mice (Figure 7), and that PAPPA2 is expressed in areas that show a high density of CCK2R-expressing ECL cells in patients with type 1 gNETs (Figure 2*C* and *D*). Gastrin also directly increased PAPPA2 expression in a human gastric epithelial cell line (Figure 4) and in primary mouse gastroid cultures (Figures 5 and 6), again in a CCK2R-dependent manner. One limitation of our experimental approach, however, was that unlike patients with autoimmune atrophic gastritis, normal mouse gastroids that have been cultured in the absence of immortalized stomach mesenchymal cells are enriched for the stem cell niche and contain very few differentiated cells such as ECL cells.^28^ Thus, other CCK2R-expressing cells also may be involved in regulating PAPPA2 expression in this mouse gastroid model system. Moreover, inhibition of PAPPA2 expression partly reversed gastrin-induced changes in cell migration and cellular remodeling in AGS_GR_ cells (Figure 8). These effects appear to occur as a result of increased IGF bioavailability, and altered IGFBP-3 cleavage appears to contribute (Figure 9). Thus, gastrin directly increases PAPPA2 expression in CCK2R-expressing cells in the stomach and this phenomenon appears to be functionally important.

Pregnancy-associated plasma protein-A2/PAPPA2 is an IGFBP proteinase.^24^ It is highly expressed in the placenta and in other tissues such as the gall bladder, kidney, and stomach at much lower levels.^29^ PAPPA2 and its homologue PAPPA^30^ are the only 2 members of the pappalysin family within the metzincin superfamily of metalloproteinases,^31^ which also includes the MMPs and a disintegrin and metalloproteinase family of enzymes. All metzincins share the elongated zinc-binding motif (HEXXHXXGXXH), but PAPPA and PAPPA2 are relatively large proteins and contain modules that are not present in other metzincins, such as the Lin-Notch repeat module, which determines PAPPA substrate specificity.^32^ The pappalysins also are distinct from other proteases such as the MMPs in that they do not cleave matrix proteins. The only known substrates of PAPPA and PAPPA2 are subsets of the IGFBPs.^30^ Proteolysis of IGFBPs increases the availability of bioactive IGF to activate the IGF-I receptor. Aberrant IGF signaling has been shown to be associated with cancer development at several sites.^33^

*H pylori* infection and hypergastrinemia have been shown previously to alter gastric IGF signaling and promote gastric tumor development via a related mechanism. Both factors have been shown to stimulate MMP7 secretion by gastric epithelial cells. This MMP7 in turn cleaves IGFBP-5, leading to increased IGF2 bioavailability from subepithelial myofibroblasts. IGF2 acts on both gastric epithelial and stromal cells to alter the gastric microenvironment and promote tumor development.^10^, ^11^, ^12^, ^13^, ^14^, ^15^ IGFBP-5 up-regulation also was observed in approximately 50% of gastric cancers.^34^ Tissue inhibitors of metalloproteinases (TIMPs), specifically TIMP1, TIMP3, and TIMP4, have been shown to inhibit this process.^35^ However, TIMPs do not inhibit the effects of pappalysins.

Our data suggest that in addition to this previously described MMP7-associated mechanism, hypergastrinemia also stimulates the secretion of PAPPA2 by gastric epithelial cells. The cells involved express CCK2R and in the setting of autoimmune atrophic gastritis these are most likely to be ECL cells. However, paracrine signaling mechanisms involving non-CCK2R–expressing cells also may be involved. Secreted PAPPA2 selectively cleaves IGFBPs in the stomach, resulting in altered IGF signaling and consequent alterations to important tumor-associated parameters such as cell migration. The mechanism is crucially different, however, from that induced by MMP7, because the IGFBP family member that is cleaved predominantly by PAPPA2 appears to be IGFBP-3. It therefore is likely that both MMP7 and PAPPA2 lead to contributions to gastrin-induced increased IGF bioavailability in the stomach, particularly because the responses to gastrin shown in Figure 9 were inhibited completely by the IGF-1 antagonist AG1024, but only partially inhibited by PAPPA2 siRNA.

In conclusion, our findings suggest the presence of a novel gastrin-regulated signaling pathway that appears to be important during type I gNET development. Moreover, inhibition of this pathway by netazepide appears to be one mechanism by which this drug results in gNET tumor regression. It therefore will be interesting to investigate whether single-nucleotide polymorphisms in the PAPPA2 gene influence gNET pathogenesis or netazepide response. In addition, it also will be important to investigate whether this pathway also plays an important role in *H pylori*–associated gastric carcinogenesis, particularly because the premalignant condition of atrophic gastritis also is associated with hypergastrinemia.

## Methods

### Materials

Amidated unsulfated heptadecapeptide gastrin (G17) was from Bachem, YM022 was from Tocris Bioscience (Abingdon, UK), and netazepide was a gift from Trio Medicines, Ltd (London, UK). All other routine supplies were from Sigma (Gillingham, UK) unless otherwise stated.

### Human Samples

Serum and gastric corpus biopsy specimens were taken with informed consent and ethical approval at several time points from 8 hypergastrinemic patients with type 1 gNETs who were enrolled in studies 1 and 2 of a phase 2 clinical trial involving treatment with netazepide, as previously described.^22^^,^^23^ Control samples were obtained with ethical approval from 10 patients who had a normal upper gastrointestinal endoscopy, normal gastric histology, no histologic or serologic evidence of *H pylori* infection, were not taking proton pump inhibitor drugs, and who had fasting serum gastrin concentrations less than 40 pmol/L, as previously described.^36^

### Mouse Samples

All mouse experiments were performed under UK Home Office project license approval. Animals were housed in a specific pathogen-free facility at the University of Liverpool with access to food and water ad libitum. Gastric mucosal scrapes were taken from 15- or 33-week-old transgenic hypergastrinemic INS-GAS mice on a FVB/N genetic background^26^^,^^37^ and from wild-type FVB/N mice, and stored in RNAlater (Sigma, Gillingham, UK) solution for gene expression analysis. In addition, whole stomachs were formalin-fixed and paraffin-embedded for histology.

### Cell Culture

**Figure 10:**
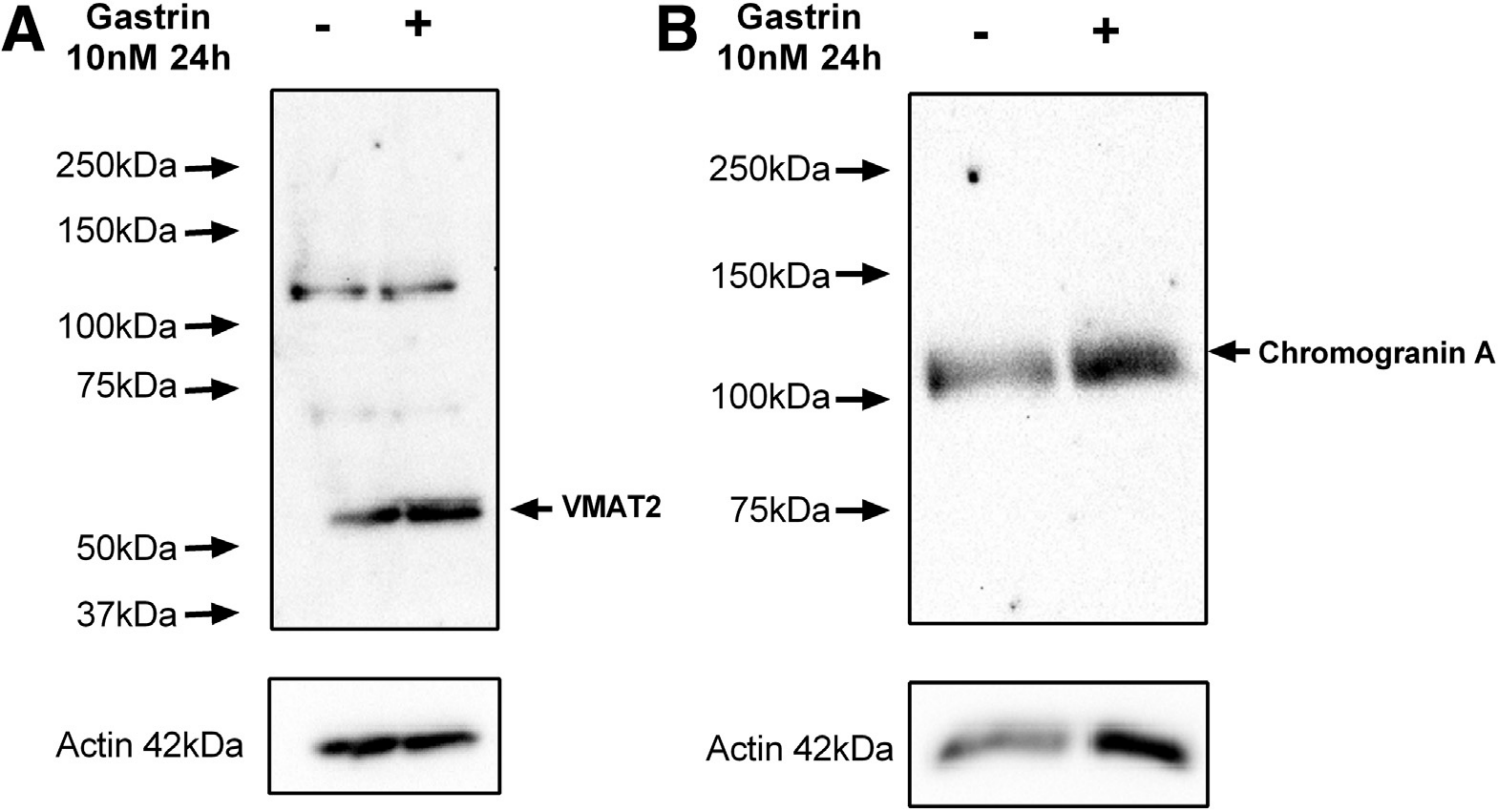
**Western blot for (*A*) VMAT2 and (*B*) chromogranin A in AGS_GR_ cells treated with and without 10 nmol/L gastrin for 24 hours**.

The human AGS_GR_ gastric adenocarcinoma cell line that stably expresses human CCK_2_R^27^ was cultured in nutrient mixture F-12 Ham’s medium supplemented with 10% fetal bovine serum (Gibco, Fisher Scientific, Loughborough, UK), 2 mmol/L L-glutamine, and 1% combined antibiotics streptomycin and penicillin. AGS_GR_ cells expressed chromogranin A and VMAT2 proteins, and the abundance of both proteins increased after gastrin treatment (Figure 10). The L-WRN cell line that secretes Noggin, R-spondin-3, and Wnt-3a into the medium was purchased from American Type Culture Collection (ATCC CRL-3276) and was cultivated in phenol-red free Dulbecco’s modified Eagle medium supplemented with 10% fetal bovine serum (Gibco), 2 mmol/L L-Glutamine, 0.5 mg/mL genetecin (Gibco), and 0.5 mg/mL hygromycin B (Invitrogen, ThermoFisher Scientific, UK). L-WRN cells were used to generate growth factor–containing conditioned media for gastroid culture. All cells were maintained in a humidified atmosphere of 5% CO_2_/95% O_2_ in Galaxy R (Wolf Laboratories, York, UK) incubators at 37°C, and AGS_GR_ cells underwent antibiotic selection with 2 μg/mL puromycin for 7 days before experimentation.

### siRNA Transfection

AGS_GR_ cells were transfected with SMARTpool ON-TARGET*plus* human PAPPA2 siRNA (Horizon Discovery, UK) or SMARTpool ON-TARGET*plus* nontargeting Pool siRNA (Horizon Discovery) (Table 1) for 48 hours according to the manufacturer’s instructions and using DharmaFECT 1 transfection reagent (GE Dharmacon, Lafayette, CO). Cell culture medium then was changed to serum-free medium when 10 nmol/L gastrin treatment was applied.

**Table 1:** Primer and siRNA Sequences

Target gene	Type	Sequence
*PAPPA2*	Forward primer	GCATCTCAGCTGTGGCTCTA
*PAPPA2*	Reverse primer	AGTTACTGGGAGCCGAAAGAC
*GAPDH*	Forward primer	CAGCAAGAGCACAAGAGGAA
*GAPDH*	Reverse primer	GTGGTGGGGACTGAGTGT
*PAPPA2*	siRNA pool	CAUCAUCGCAGGUGUGUUU GCCCAAGCAUUCCCUUAAA GGGCUCCGUUCACCAACUA CAAGAGGGCAUACAUGAGU
Nontargeting (scrambled)	siRNA pool	UGGUUUACAUGUCGACUAA UGGUUUACAUGUUGUGUGA UGGUUUACAUGUUUUCUGA UGGUUUACAUGUUUUCCUA

### Gastric Organoid (Gastroid) Culture

The stomachs of 12-week-old C57BL/6 or INS-GAS mice were removed and washed in ice-cold phosphate-buffered saline (PBS). After removing the forestomach the remaining tissue was cut into 2-cm × 1-cm sections and placed into ice-cold chelation buffer (5 mmol/L EDTA in PBS) for 2 hours at 4°C with constant agitation. Glands were released in shaking buffer (43.3 mmol/L sucrose, 59.4 mmol/L sorbitol in PBS) and approximately 5000 glands were resuspended in 500 μL phenol-red free Matrigel (Scientific Laboratory Supplies, Nottingham, UK) containing 50 ng/mL epidermal growth factor, 100 ng/mL fibroblast growth factor 10 (from R&D Systems, Abingdon, UK), and 10 nmol/L G17. Matrigel (50 μL) was plated in wells, using frozen pipette tips, of a prewarmed 24-well plate. Glands were incubated for 20 minutes at 37°C before application of 500 μL basal medium containing 50% L-WRN conditioned medium and 50% Dulbecco’s modified Eagle medium/F12 medium containing 2% L-glutamine, 20 mmol/L HEPES, 2% N2, and 4% B27 supplements with 2% primocin and maintained at 5% CO_2_/95% O_2_ in Galaxy R incubators at 37°C. Medium was replaced every 4 days with fresh growth medium, which is basal medium plus epidermal growth factor (50 ng/mL), fibroblast growth factor 10 (100 ng/mL), and G17 (10 nmol/L). Gastroids were passaged every 5–7 days. Experiments involving INS-GAS mice used gastroids that had been cryopreserved previously.

### Treatment of Gastric Organoids

**Figure 11:**
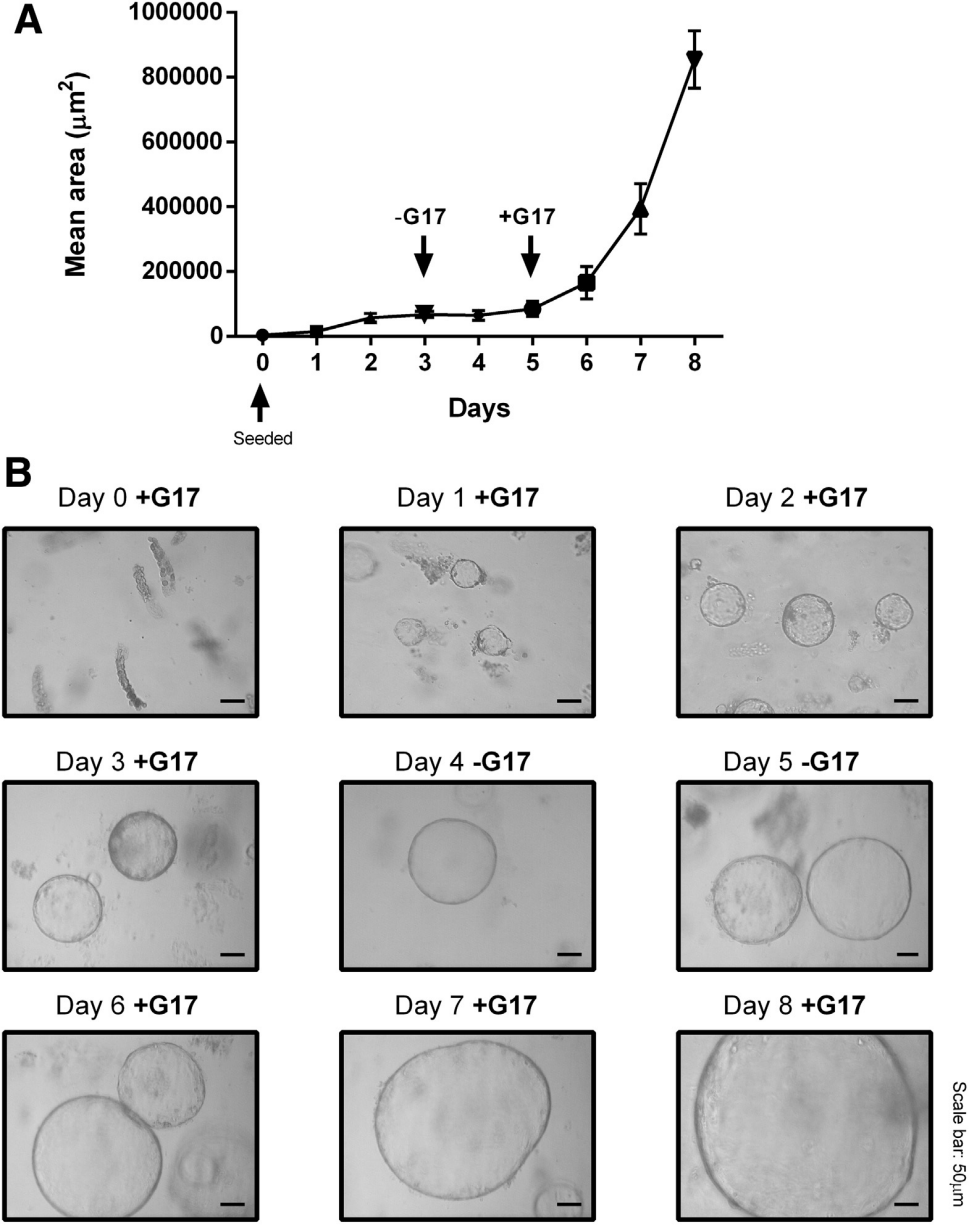
**Organoids retain cell viability after removal of G17 for 48 hours, once G17 is reapplied.** Organoids increased in area until the removal of 10 nmol/L G17 at day 3, when growth was inhibited. (*A*) When 10 nmol/L G17 then was reapplied at day 5, gastric murine organoid area increased, time-dependently. (*B*) Representative images are shown.

Organoids were split after 5–7 days of growth in 50 μL Matrigel-containing growth factors and left to grow in basal media. Gastrin was removed 3 days after passage for 24 hours before treatment. Gastroids retained viability after withdrawal of gastrin from the culture media for up to 48 hours (Figure 11). Organoids initially were treated with 0–10 nmol/L gastrin for 6–24 hours to determine a dose response. Then, organoids were pretreated for 20 minutes with or without YM022 or netazepide (100 nmol/L), followed by 10 nmol/L G17 for 6–24 hours. Untreated organoids and treatments of dimethyl sulfoxide (1%) or 10 nmol/L G17 alone were used as negative, vehicle, and positive controls, respectively. After treatment, 5 images per well were taken using a Zeiss Axiovert 25 microscope (Carl Zeiss Microscopy) at ×10 magnification and the organoid area was calculated using ImageJ software (National Institutes of Health, Bethesda, MD). Organoids then were washed with ice-cold PBS and harvested using 400 μL cell dissociation solution for 1 hour on ice with constant agitation. Whole organoids were pelleted and stored at -80°C before RNA extraction.

### RNA Isolation and Reverse Transcription

RNA was extracted from human biopsy specimens using Tri-Reagent as previously described.^22^ Total RNA was isolated from cells and mouse tissues using the RNeasy Mini Kit (Qiagen). Eluted RNA was reverse-transcribed into complementary DNA using the miScript RT II Kit (Qiagen) according to the manufacturer’s handbook and stored as undiluted complementary DNA at -20°C before real-time PCR.

### Microarray

Samples were hybridized onto the Affymetrix Human Gene 2.0 ST arrays, which provided coverage of more than 30,000 coding transcripts and more than 11,000 long intergenic noncoding transcripts according to the manufacturer’s instructions. Briefly, 200 ng RNA was prepared using the Affymetrix GeneChip Wild-Type Plus reagent kit and 3.5 ug of fragmented and labeled single-stranded DNA was loaded onto the array. Arrays were hybridized for 16 hours at 45°C at 60 rpm in the Affymetrix hybridization oven 640, then washed and stained on the GeneChip Fluidics station 450 using fluidics script FS450_0002. Arrays were scanned using the Affymetrix GeneChip scanner 3000 7G and analyses were obtained using the Affymetrix GeneChip Command Control and Expression Console software.

The detection of significant KEGG pathways was conducted using R package gage.^38^ The log_2_ fold change from baseline vs week 6 contrast was inputted into gage for significance testing. The default settings were used, except that same.dir=FALSE was selected to consider both up-regulation and down-regulation together. Significant pathways were detected using the criterion of false-discovery rates less than 5%. Data from the microarray analysis can be found at ArrayExpress using accession number E-MTAB-6473.

### qPCR

Human PAPPA2 forward and glyceraldehyde-3-phosphate dehydrogenase qPCR primer pair sequences were purchased from Eurogentec (Table 1). Gene expression was assessed using Quantitect primer assays (Qiagen) with SYBR green. Glyceraldehyde-3-phosphate dehydrogenase was used for normalization, according to the Quantitect Primer Assay Handbook (Qiagen), and samples were run in a real-time LightCycler 480 (Roche). Each sample was run in quadruplicate and analysis used the ΔΔC_T_ method for relative quantification.

### Cell Migration Assays

Transfected or untransfected AGS_GR_ cells were grown as confluent monolayers in 24-well plates before a cell-free region was created using a 2-μL pipette tip. Cells were washed twice in PBS, then washed twice in serum-free media before the treatment was applied. Whole cells that had migrated into the denuded region were counted and scratch wound width was measured using a graticule at 0 and 8 hours after treatment. Representative images were taken at these times using a Zeiss Axiovert 25 microscope (Carl Zeiss Microscopy).

### Cell Morphology Assays

Transfected or untransfected AGS_GR_ cells (1 × 10^4^/well) were treated with or without 10 nmol/L gastrin for 6 hours. After treatment, cells were fixed using 3:1 methanol:acetic acid and stained with 0.3% crystal violet. The number of cells that presented long processes were counted as a percentage of total cells in 3 reference fields (>100 cells) per treatment and representative images were taken using the Zeiss Axiovert 25 microscope (Carl Zeiss Microscopy).

### Immunofluorescence

Transfected or untransfected AGS_GR_ cells were seeded onto 13-mm diameter coverslips (VWR International Ltd) in 24-well plates and left to adhere for 24 hours. Mechanically disrupted gastroids also were seeded onto coverslips in 24-well plates and left to adhere for 3 days. Cells and 2-dimensional gastroid monolayers were pretreated with or without CCK2R inhibitors and with or without 10 nmol/L gastrin for 24 hours.

After treatment, samples were washed with PBS, fixed with 4% paraformaldehyde for 30 minutes, and permeabilized with 0.2% phosphate buffered Triton-X (0.03 g bovine serum albumin [BSA], 10 mL PBS, and 20 μL Triton-X 100; Sigma, Gillingham, UK) for 30 minutes.

For immunofluorescence, samples were blocked in 10% swine serum (Agilent Technologies, Stockport, UK) for 45 minutes at room temperature before overnight incubation with rabbit polyclonal anti-Plac3 (PAPPA2) primary antibody (Thermo Fisher Scientific, UK) diluted 1:500 in PBS in a humidified chamber at 4°C. Salt washes were applied before incubation with swine anti-rabbit fluorescein isothiocyanate–conjugated secondary antibody (Agilent Technologies) diluted 1:500 in 1% BSA in PBS for 1 hour, protected from light. Samples were washed before mounting with Vectashield mounting media with 4′,6-diamidino-2-phenylindole (Vectorlabs, Peterborough, UK) onto glass slides for visualization. Images were captured using the Olympus BX51 fluorescence microscope (Olympus, South-End-on-Sea, UK) at 6 reference fields (>100 cells) per treatment, and relative intensities of nuclear and cytoplasmic staining were analyzed using AxioVision Rel. 4.8 software (Carl-Zeiss, White Plains, NY). Samples stained with secondary antibody alone were used as nonspecific binding controls.

### Immunohistochemistry

Immunohistochemistry was performed on 4-μm–thick formalin-fixed, paraffin-embedded human gastric biopsy sections. Subsequent to deparaffinization, antigen retrieval was performed by microwaving in 10 mmol/L citric acid buffer (pH 6) for 20 minutes for chromogranin A immunohistochemistry only. Endogenous peroxidase activity and nonspecific binding were blocked at 22°C using a peroxidase block (Dako) for 5 minutes and protein block (Dako) for 30 minutes, respectively. Tissue sections were incubated with monoclonal mouse primary PAPPA2 antibody (PA257^39^) or polyclonal rabbit primary chromogranin A antibody (Santa Cruz Biotechnology, Heidelberg, Germany) diluted in 50 mmol/L Tris, 100 mmol/L NaCl, 1 mmol/L CaCl_2_, 1% BSA, pH 7.4, for 1 hour at 22°C, followed by horseradish peroxidase–conjugated goat anti-mouse or anti-rabbit immunoglobulins (Dako) for 30 minutes at 22°C, and finally developed by incubation with 3,3′-diaminobenzidine tetra hydrochloride. Immunostained tissues were counterstained with Mayer’s hematoxylin (VWR International Ltd). Separate mouse tissue sections were stained with H&E (VWR International Ltd) and scored for quantitative histologic assessment as previously described.^40^

### Enzyme-Linked Immunosorbent Assays

PAPPA2 concentrations in serum samples were determined essentially as previously reported using an assay based on 2 monoclonal antibodies and calibration of the assay with recombinant human PAPPA2.^39^

### Gastrin Radioimmunoassay

Serum gastrin concentration was measured by radioimmunoassay using antiserum L2 directed against the C-terminus of all amidated gastrins as previously described.^41^^,^^42^ Normal fasting circulating gastrin concentrations are less than 40 pmol/L.

### Western Blot

Media samples from AGS_GR_ cells were concentrated using StrataClean resin (Agilent Technologies, Stockport, UK) and processed as previously described.^7^ To assess proteolytic activity, proteins were separated on 15% sodium dodecyl sulfate–polyacrylamide gels and probed using either polyclonal goat IGFBP-3 or IGFBP-5 primary antibodies (R&D Systems) at 1:500 followed by rabbit anti-goat horseradish peroxidase–conjugated secondary antibody at 1:5000 (R&D Systems). Membranes were developed using Supersignal (Thermo Fisher) and chemiluminescence was detected using a Bio-Rad ChemiDoc XRS+ (Bio-Rad). Densitometry was performed using ImageLab software (version 3.0; Bio-Rad, Hertfordshire, UK).

### Statistics

Data are presented as the means or percentage of control ± SEM. Either 1- or 2-way analysis of variance, as appropriate, with the Sidak post hoc test were used to establish statistical significance. *P* < .05 was considered significant. A Mann–Whitney *U* test was used to assess statistical differences between healthy and gNET patient samples. A Wilcoxon signed-rank test with Bonferroni correction was used to determine significant differences between repeated samples from the same patients, and *P* < .0125 was considered significant after correction.
